# Morphological characteristics of the left gastric, common hepatic and splenic arteries. A descriptive study in human cadaveric specimens

**DOI:** 10.1590/0100-6991e-20233403-en

**Published:** 2023-01-24

**Authors:** BLADIMIR SALDARRIAGA, OSCAR LARROTTA, LUIS BALLESTEROS

**Affiliations:** 1 - Universidad Autónoma de Bucaramanga, Basic Science - Bucaramanga - Santander - Colômbia; 2 - Universidad Industrial de Santander, Basic Science - Bucaramanga - Santander - Colômbia

**Keywords:** Anatomic Variations, Hepatic Artery, Splenic Artery, Gastric Artery, Variação Anatômica, Artéria Hepática, Artéria Esplênica, Artéria Gástrica

## Abstract

**Objective:**

to evaluate the morphology of the branches of celiac trunk (CT), left gastric (LGA), common hepatic (CHA), and splenic (SA) arteries in cadaveric specimens from a sample of a Colombian population.

**Methods:**

descriptive cross-sectional study of 26 blocks from the abdominal upper segment of human cadavers who underwent forensic autopsies at the Instituto de Medicina Legal at Bucaramanga, Colombia. The vascular beds of the celiac trunk were, subsequently, perfused with a semi-synthetic resin.

**Results:**

the diameters of LGA, CHA, and SA were 3.6±0.8mm, 5,2±1.2mm, and 5.9±1.0mm, respectively. Statistically, LGA and SA were different (p=<0.001). SA followed a linear trajectory in 8 (31%) samples, slightly tortuous in 4 (15%), and tortuous in 14 (54%). The tortuosity index was 1.25±0.18. Of the branches of CHA, the proper hepatic artery (PHA) had 4.8±1.2mm in diameter and 18.8±9.1mm in length, whereas the gastroduodenal artery (GDdA) had 4.1±0.8mm. In 2 cases (7.7%), an accessory hepatic artery from the LGA was found to supply perfusion to the left hepatic lobe. Finally, in 2 cases (7.7%) the SA came independently from the abdominal aorta.

**Conclusion:**

the observed emergence incidence of the CT branches from the same level as reported in the literature is lower. The characterization, along with their variants, of LGA, CHA, and SA must be considered in surgical procedures in the upper abdominal segment, to avoid iatrogenic complications.

## RESUMO

**Objetivo:**

avaliar a morfologia dos ramos das artérias do tronco celíaco (CT), gástrica esquerda (LGA), hepática comum (HC) e esplênica (SA) em espécimes cadavéricos de uma amostra de uma população colombiana.

**Métodos:**

estudo transversal descritivo de 26 blocos do segmento superior abdominal de cadáveres humanos submetidos a necropsias forenses no Instituto de Medicina Legal de Bucaramanga, Colômbia. Os leitos vasculares do tronco celíaco foram, posteriormente, perfundidos com resina semissintética.

**Resultados:**

os diâmetros de LGA, CHA e SA foram 3,6±0,8mm, 5,2±1,2mm e 5,9±1,0mm, respectivamente. Estatisticamente, LGA e SA foram diferentes (p=<0,001). A SA seguiu uma trajetória linear em 8 (31%) amostras, levemente tortuosa em 4 (15%) e tortuosa em 14 (54%). O índice de tortuosidade foi de 1,25±0,18. Dos ramos do ACS, a artéria hepática própria (APH) tinha 4,8±1,2mm de diâmetro e 18,8±9,1mm de comprimento, enquanto a artéria gastroduodenal (GDdA) tinha 4,1±0,8mm. Em 2 casos (7,7%), uma artéria hepática acessória do GIG foi encontrada para fornecer perfusão ao lobo hepático esquerdo. Finalmente, em 2 casos (7,7%) a SA veio independentemente da aorta abdominal.

**Conclusão:**

a incidência observada de emergência dos ramos de TC do mesmo nível relatado na literatura é menor. A caracterização, juntamente com suas variantes, de LGA, CHA e SA deve ser considerada em procedimentos cirúrgicos no segmento abdominal superior, para evitar complicações iatrogênicas.

## INTRODUCTION

Usually, the celiac trunk (CT), the main branch of the abdominal aorta (AA) branches out into the left gastric artery (LGA), splenic artery (SA), and common hepatic artery (CHA), with several variants described in the literature, especially, the true trifurcation (“tripus Halleri”) in 22-30% of the cases^1^ ^-^ ^3^, or bifurcated presentation, called hepatogastric, gastrosplenic, hepatosplenic^4^ ^,^ ^5^. The LGA branches out from the AA^6^ in 2.5-13.1%^3^ ^,^ ^7^, is the first branch of the CT^8^ in a 68% of the cases, with a usual diameter of 3.8-5mm^1^ ^,^ ^2^ ^,^ ^9^. In 0.5-11%^8^ ^,^ ^10^, the accessory hepatic artery (AcHA) is a branch of the LGA^8^, has the same origin as the left hepatic artery (LHA) in 0.16%-15% of the cases^10^ ^,^ ^11^.

Usually, the CHA originates from the CT (94-96.3% of the cases)^11^ ^,^ ^12^, while other origin variants of the CHA have been reported, such as superior mesenteric artery (SMA) (0.6-13.1%)^10^ ^,^ ^11^ ^,^ ^13^; and, from the AA (0.3-0.6%)^11^ ^,^ ^15^, of the LGA^16^ in 1.1%, there is an absence of CHA, with an independent origin of the gastroduodenal artery (GDdA) and the proper hepatic artery (PHA) was branching out from the CT, while in the 10% the CHA divided in GDdA and PHA, where the left branch is replaced with a branch of the LGA^15^. The length of the CHA is 24.2mm and its diameter is 6.2mm^12^. The knowledge of the origin, trajectories, and branches of the hepatic arteries is important for hepatic and biliary surgical procedures such as liver transplantation and aneurysm approach^12^ ^,^ ^14^ ^,^ ^17^.

The SA has the biggest diameter (6.1mm)^2^, and its length is 173 mm. It is the first branch of the CT in 8.1%^1^. Its distal segment, usually at the splenic hilum, bifurcates in 80% of the cases and trifurcates in 20%^11^. One of its particular morphological features is its tortuosity which increases with aging and pathological conditions such as atherosclerosis and arterial hypertension^18^ ^,^ ^19^.

Considering the variability of the arterial branches of the CT, their radiologic and surgical complexity, and the scarcity of anatomical information on these vessels, with limited populational descriptions of dissection, perfusion, or imaging studies, with a clear basic-clinical orientation^12^ ^,^ ^15^ ^,^ ^18^, this study provides relevant and reference anatomical information on the morphology of LGA, CHA, and SA in a sample of Colombian individuals, which can be used in medical areas such as surgical procedures. 

## MATERIALS AND METHODS

This cross-sectional descriptive and non-probabilistic study determined the anatomical features of the celiac trunk branches in 26 blocks of the supra-mesocolic bed (liver, stomach, pancreas, spleen, abdominal aorta) from non-reclaimed male mixed-raced adult cadavers (20 to 65 years old), from autopsies at the National Institute of Legal Medicine and Forensic Sciences, Northeast Regional, at Bucaramanga, Colombia. Exclusion criteria were trauma, illness, or surgical approaches near the time of death that compromised the organs and their vascular structures from the upper abdomen. This study was reviewed and approved by the Ethics Committee of the Universidad Autónoma de Bucaramanga (UNAB).

A 14-gauge catheter was placed onto the proximal stump from the abdominal aorta (AA) and fixed with a silk suture, while the distal aortic stump and the origin of its lumbar emergent branches were fixed (AA is bound proximally to its bifurcation) with a 2/0 silk suture; followed by the cleaning of the vascular beds with physiologic saline solution to eliminate clots and hematic detritus. Then, the AA and the CT branches were perfused with a red-dyed polyester semi-synthetic resin (Mixture of GP40L 83%, Cobalt 1%, methyl ethyl ketone 1% and styrene 15%, in a controlled environment of 25°C). 

The resin injection (250cc, with 14-gauge syringe) was stopped once the positive pressure was verified at the syringe plunger level, meaning that all the studied structures were filled. Consecutively, the abdominal blocks were fixed for 15 days with Formaldehyde 10%.

After fixation, the CT branches were dissected from their origin to their distal segments. Then, using a digital caliper (Mitutoyo^®^) their external diameter was measured at 0.5mm from each origin. Finally, the qualitative features of the different morphological expressions of each branch were recorded. A straight linear trajectory was registered when there was any curvature above the pancreatic upper edge; a slightly tortuous trajectory if mild and tortuous upper and lower ripples were found. Furthermore, the tortuosity index (TI)^19^ was calculated in addition to a photographic record of the samples.

### Statistical analysis

Using Microsoft Excel^®^ (Microsoft Corporation, 2019) data were registered and continuous variables are expressed as mean and SD or median and interquartile ranges. Categorical variables were expressed as frequencies. For the statistical analysis, using SigmaPlot 14.5, the t-student test was used for parametrical distribution, and the Mann-Whitney test for non-parametrical, with a statistical significance of p<0.05. Finally, a test for multiple correlations for independent variables using the Spearman method to calculate Rho (correlation coefficient) for associations with p<0.05 was performed.

## RESULTS

**Figure 1:**
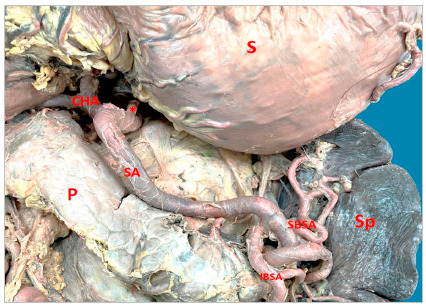
Celiac trunk hepatosplenic. Winding splenic artery. CHA: Common hepatic artery; *Left gastric artery; P: Pancreas; IBSA: Inferior branch splenic artery; SBSA: Superior branch splenic artery; S: Stomach; Sp: Spleen.

**Figure 2:**
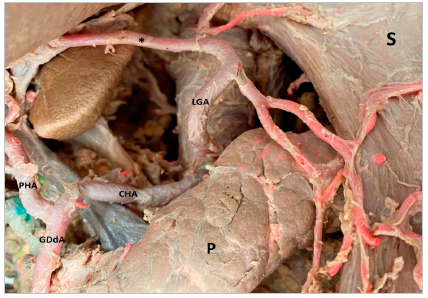
Left gastric artery originating directly from the abdominal aortic artery. CHA: Common Hepatic artery; GDdA: Gastroduodenal artery; LGA: Left Gastric artery; P: Pancreas; PHA: Proper Hepatic artery; S: Stomach; Sp: Spleen; *accessory hepatic artery originating from left gastric artery.

**Figure 3:**
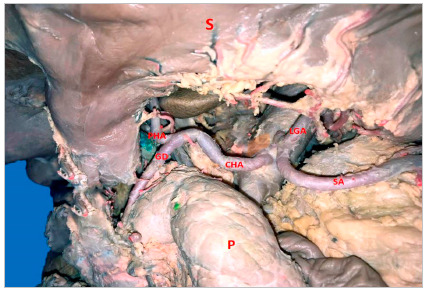
Common hepatic artery originating from the abdominal aortic artery. CHA: Common Hepatic Artery; GDdA: Gastroduodenal Artery; LGA: Left Gastric Artery; P: Pancreas; PHA: Proper Hepatic artery; SA: Splenic Artery; S: Stomach; Sp: Spleen.

**Figure 4:**
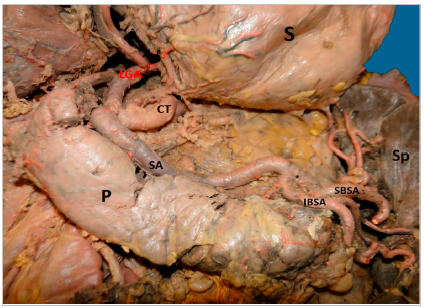
Splenic artery of tortuous trajectory. CT: Celiac Trunk; P: Pancreas; SA: Splenic Artery; IBSA: Inferior Branch Splenic Artery; SBSA: Superior Branch Splenic Artery; Sp: Spleen; S: Stomach.

**Figure 5:**
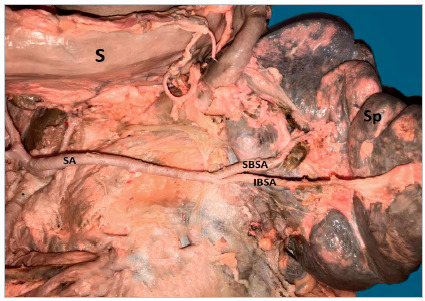
Splenic artery of straight trajectory. IBSA: Inferior Branch Splenic Artery; SA: Splenic Artery; SBSA: Superior Branch Splenic Artery; S: Stomach; Sp: Spleen.

In 16 (61.5%) cases the CT was bifurcated (hepatosplenic) with the left gastric artery (LGA) emerging next to the common hepatic artery (CHA) and splenic artery (SA) (Figure 1); in 7 samples (26.9%) the CHA, SA, and LGA branched out at the same level, while the CT hepatosplenic (LGA originates from the AA) (Figure 2) and gastrosplenic (CHA branches out from the AA) (Figure 3) types were observed in 3.8% of the samples. The SA (Figures 4 and 5) length was 92.5±16.9mm with no significant differences from the other branches, whereas its diameter (5.9±1.0) was statistically significant (p<0.001) from the LGA (Table 1).

**Table 1:** Length and diameter of the branches of the Celiac Trunk.

	x	DE	MAX	MIN	MEDIANA
SA-L	92.5	16.9	130.7	68.0	88.5
SA-D	5.9	1.0	8.6	4.2	5.6*
CHA-L	26.7	11.0	53.6	8.0	24.8
CHA-D	5.2	1.2	7.6	3.1	5.4
LGA-L	30.1	8.8	46.7	12.7	28.7
LGI-D	3.6	0.8	5.2	2.3	3.5

CHA-L: Common hepatic artery length; CHA-D: Common hepatic artery Diameter; LGA-L: Left gastric artery length; LGI-D: Left gastric artery diameter; SA-D: Splenic artery diameter; SA-L: Splenic artery length, Measurements are given in mm. *Statistical significance p<0.001 (t-student).

**Figure 6:**
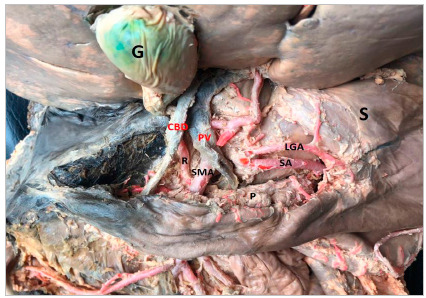
Right hepatic artery originated from the superior mesenteric artery. CBD: Comon bile duct (Ductus choledochus); G: Gallbladder; LGA: Left gastric artery; P: Pancreas; R: Right hepatic artery; SA: Splenic artery; SMA: Superior mesenteric artery; S: Stomach. Note: The pancreas was displaced downward and the portal vein to the left.

From the branches of the CHA, the diameter of the proper hepatic artery (PHA) was 4.8±1.2mm, while the diameter of the gastroduodenal artery (GDdA) was 4.1±0.8. Table 2 shows the morphometric data of the CHA, SA, and LGA collateral vessels. In 2 cases, the accessory hepatic artery (AcHA) was branched from the LGA (AcHA-LGA) giving irrigation to the left hepatic lobe. In one case (3.8%) the right hepatic artery originated from the superior mesenteric artery, in a retropancreatic position (Figure 6). 

**Table 2:** Length and diameter of the collaterals of the branches of the celiac trunk..

	x	DE	MAX	MIN	MEDIANA
SBSA-D	4.2	0.9	5.5	2.3	4.5
IBSA-D	3.6	1.1	5.1	2.0	3.6
HPA-L	18.8	9.1	33.3	7.5	19.1
HPA-D	4.8	1.2	7.7	3.1	4.4
RHA-D	4.1	1.2	6.6	2.0	4.1
RHA-L	43.5	13.1	60.6	24.5	41.4
LHA-D	3.2	0.8	4.7	1.7	3.1
LHA-L	31.7	10.8	49.1	16.9	31.8
AcHA-D-LGA	2.1	0.2	2.2	2.0	2.1
GDdA-D	4.1	0.8	5.7	2.6	4.1
GDdA-L	25.7	10.1	32.3	10.8	29.9
RGA-D	3.2	1.0	4.1	2.2	3.2

AcHA-D-LGA: Accessory hepatic artery diameter of left gastric artery; GDdA-D: Gastroduodenal artery diameter; GDdA-L: Gastroduodenal artery length; LHA-D: left hepatic artery diameter; LHA-L: left hepatic artery length; PHA-L: Proper hepatic artery length; PHA-D: Proper hepatic artery diameter; RHA-D: Right hepatic artery diameter; RHA-L: Right hepatic artery length; RGA-D: Right gastric artery diameter; SBSA-D: Superior branch of the splenic artery diameter; IBSA-D: Inferior branch of the splenic artery diameter; Data are given in mm.

The SA had a linear straight trajectory (Figure 5) in 8 samples (31%), slightly tortuous (Figure 3) in 4 samples (15%), and frankly tortuous (Figures 1 and 4) in 14 samples (54%). The calculated TI was 1.25±0.18, with no significant differences among straight and tortuous trajectories. The statistical hypothesis for the TI with n terminal branches (at the splenic hilum) using the Mann-Whitney test yielded statistically significant differences in the variable “number of branches of the upper branch of SA” (p<0.001), the variable “number of branches of the lower branch of SA” (p=0.009) and “total of branches” (p<0.001); and, using Spearman correlation test, the TI only was negatively correlated with the number of branches of the upper branch of SA (Rho=-0.57; p=0.032). Additionally, there was a positive correlation between the tortuosity index and the CT diameter (CT-D) (Rho=0.55; p=0.029).

The SA, entering the splenic hilum, was bifurcated in upper and lower branches (Figures 4 and 5), each dividing into two or three branches as well. For the variable number of branches of the upper branch of SA, 29% of the simples had 3 branches, and 71%, had two branches, while for the lower branch of SA, 14% of samples had 3 branches, 64% had two branches, and in 21%, just one branch.

From the correlation analysis, there were positive associations among splenic artery length (SA-L) and “Number of branches of the upper branch of the SA”, “common hepatic artery length (CHA-L)”, “left gastric artery length (LGA-L)”, and “left hepatic artery diameter (LHA-D)”, as well for “common hepatic artery diameter (CHA-D)” with “diameter of the celiac trunk”, “spleen artery diameter (SA-D)”, “lower branch of the splenic artery diameter (LBSA-D)”, and “number of branches of the SA upper branch”. Besides, there were more positive correlations among “gastroduodenal artery diameter (GDdA-D)” with “diameter of the celiac trunk (CT-D)”, “diameter of the lower splenic artery branch”, “common hepatic artery length (CHA-L)”, “common hepatic artery diameter (CHA-D)”, “left gastric artery diameter (LGA-D)”, and “right hepatic artery diameter (RHA-D)”. On the other hand, a negative correlation was found between “proper hepatic artery length (PHA-L)” and “proper hepatic artery diameter (PHA-D)”, between “right hepatic artery diameter (RHA-D)” and “left hepatic artery diameter (LHA-D)” and between “left hepatic artery diameter (LHA-D)” and “left hepatic artery length (LHA-L)”. The qualitative description of the morphologic variants of the emergent branches of CT, such as the inferior phrenic artery originated from the proximal part of the CT, the left gastric artery gives rise to the left hepatic artery, the right hepatic artery emerges from the LHA. The common hepatic artery emits the left hepatic artery and the gastroduodenal artery.

## DISCUSSION

The hepatosplenic pattern was the more frequent pattern in this study, using the perfusion of the vascular beds and the proximal emergence of the left gastric artery (LGA) from the celiac trunk (CT) (61.5%), coinciding with the findings of Marco-Clement^20^. and at a slightly lower frequency than other studies^1^ ^,^ ^2^ ^,^ ^21^. The frequency of the emergence at the same level of the three branches of the CT (26.9%) is higher than in a previous report^22^ ^,^ ^23^, while the range of expression of this morphological feature is 32.1-89.5%^20^ ^,^ ^24^. The incidence of the independent origin of the LGA and CHA from the AA^4^ ^,^ ^6^ is reported at 1.4-2.3 and 0.4-0.6, respectivelly^11^ ^,^ ^21^ ^,^ ^24^, whereas, in this report, the incidence of an AA origin is mildly higher (3.8% for each branch). On the other hand, the origin of the CHA is highly variable, according to multiple studies. From the superior mesenteric artery (SMA) (incidence, 6.7-13.1%)^15^ ^,^ ^25^, from the LGA^16^, or absent (incidence 1.1-6.6%)^15^ ^,^ ^24^. In this study, 3.8% of the right hepatic artery (RHA) originated from the SMA, in a retropancreatic position, this anatomical variant may increase the risk of injury in liver transplant^14^. 

Regarding the diameters of the CT branches, this study found the SA as the bigger branch (5.9mm), followed by the CHA (5.2mm) and the LGA (3.6mm), results that are consistent with the report of Silveira^9^. Information about the length of the CT branches is scarce, but our findings of 26.7mm for the CHA in accordance with Silveira^9^. 

Subjects with small CT branch calibers, when added to other variables such as the presence of atheroma plaques, will have as a pathophysiological feature the hypoperfusion of organs such as the liver and pancreas, which may explain the appearance of metabolic pathologies that are generated in them. 

The high variability of information regarding the origin, length, and diameters of the usual branches derived from the CT depends upon the sample size, measurement criteria, evaluation methods (direct dissection or imaging evaluation), and the biological expressions of the anatomical structures among different populational groups.

Some of the variant origins of the CT branches have clear and important implications for the surgical procedures of the upper abdominal segment. For instance, during the total gastrectomy due to gastric cancer which requires the ligature of the LGA after the emergence of the hepatic branch to avoid the hypoperfusion of the left hepatic lobe; a proximal ligature of the left hepatic branch from the left gastric artery would deteriorate the patient’s condition, even to death^10^ ^,^ ^11^ ^,^ ^16^ ^,^ ^26^.

The splenic artery (SA) is described as the first branch of CT (2.5%)^7^, but these morphological features were not observed in our study. The length of the SA was 92.5 mm, while in other studies the range fluctuates from 76.5 to 94.1mm^1^ ^,^ ^27^. Concerning the tortuosity of the SA, the results from Fataftah et al 2020^27^ report 94.1mm for the straight SA, and 155 mm for the tortuous SA, with a tortuosity index (TI) of 1.63, higher than our TI of 1.25. Silvester et al.^19^ found, in cadavers and using angiography imaging, a TI range for cadavers of 1.01-3.58, and higher in the imaging analysis (5.25 TI), with no correlation between the TI of the SA and female gender, body mass index, abdominal cavity diameter, nor age. The tortuous SA increases the difficulty during the splenectomy due to the sinuous trajectory affecting the splenic arterial trunk as well its terminal branches. If the latter are numerous, during the surgical procedure is necessary to identify and ligate them, increasing the risk of acute pancreatitis, as a post-surgical complication, for the excessive manipulation of the tail of the pancreas. Also, the TI of the SA could be helpful for the planning of the surgical embolization to prevent clots during the splenectomy^24^, he morphological characterization of SA is important due to the high frequency of aneurysms and their high mortality in case of rupture^28^.

In our study, we did not find proximal branching of the SA, but Pandey^29^, reported that this variant is present in 1.6% of the cases, with a wide variation of the number of terminal branches of the SA, as well as no ramification in 2.8% of the cases, with 2 terminal branches in 63.1%, four branches in 18.8%, six branches in 9.7%, and more than six branches in 5.6% of the cases^29^. Sahni^1^ reported the ramification of SA in two and three lobar branches in 80% and 20% of the cases, respectively, whereas this study reports the bifurcation (upper and lower branch) of the SA in its terminal end. The reported 63.1% of the cases by Pandey 2004^29^, with terminal branches of the upper and lower branches of the SA, do not agree with our findings of bifurcation and trifurcation of those branches. 

There was a positive association between the length of the SA with the length of CHA and LGA, as well as with the diameter of LHA and the number of branches of the upper branch of SA. The same positive correlation was observed between the diameter of the CHA and the diameters of the CT, SA, and the number of branches from the upper branch of the SA. Conversely, there was a negative correlation between the length of proper hepatic artery (PHA) and the diameters of LHA and RHA. This correlation evaluation, to our knowledge, has not been reported in other studies. These assessments enrich the conceptual framework for the distribution and behavior of the arteries irrigating the organs of the upper mesocolic abdominal. In the sample studied, morphological variants were found in only one of the cases, such as the inferior phrenic artery originating from the CT, the LHA originating from the LGA, in agreement with what is reported in the literature^7^ ^,^ ^13^ ^,^ ^26^.

## CONCLUSION

In male Colombian mixed-raced adult, the incidence of hepatosplenic celiac trunk and Haller’s triad is slightly lower than reported in most previous studies. The splenic artery was the largest caliber, with statistically significant difference in relation to the other emerging CT branches. As in previous studies, agenesis or low incidence of other CT branches (inferior phrenic, accessory hepatic, superior mesenteric) was observed. A negative correlation was found between the tortuosity index and the number of branches of the superior branch of the SA, a finding not reported in the literature.

For the medical and surgical fields, the knowledge of the anatomical variants of SA, CHA, LGA, and their branches is highly important in general surgery, laparoscopic surgery, and imaging procedures, especially those involving the liver, the pancreas, the stomach, and the spleen. For instance, computed tomography angiography (CTA) allows clear delineation of vascular anatomy before the scheduled surgical procedure. During the surgical planning, the surgeon would anticipate a vascular variant and could prevent iatrogenic complications, especially those related to the hypoperfusion of the organs supplied for these variants. Part of the success of the emergency procedures compromising the CT and its branches is due to the operator’s procedural expertise, based on a profound knowledge of the variability of these vessels’ origin, caliber, and trajectory. 
